# Of Young People and Internet Cafés

**DOI:** 10.3389/fpsyg.2021.603992

**Published:** 2021-09-16

**Authors:** ZhiMin Xiao, Steve Higgins

**Affiliations:** ^1^School of Health and Social Care, University of Essex, Colchester, United Kingdom; ^2^Graduate School of Education, University of Exeter, Exeter, United Kingdom; ^3^Health Statistics Group, Institute of Health Research, College of Medicine and Health, University of Exeter, Exeter, United Kingdom; ^4^School of Education, Durham University, Durham, United Kingdom

**Keywords:** China, social media, moral-technological panic, teenagers, academic performance

## Abstract

This study examines how adolescent experience in Internet cafés (known as wangba in Chinese) relates to academic attainment in urban, rural, and Tibetan schools of China. By documenting the frustrations teenagers express in their negotiations with adults surrounding access to and use of wangba and, by comparing self-reported academic standing of students from similar backgrounds with how they differ in their experience in wangba, the study finds that visiting wangba is not strongly correlated with the probability of students reporting either high- or under-achievement. While students without any experience in wangba are substantially less likely to report academic underperformance, the association disappears after matching when the logit regression model is less model-dependent and vulnerable to the problems associated with missing data. The paper concludes that visiting wangba alone is not systematically correlated with academic attainment, and that much adult anxiety concerning adolescent visit to wangba represents moral-technological panic and, offers a simplified explanation for educational problems that have deep macrosocial roots.

## Opportunities and Risks in Cyberspace

Views on what various Information and Communication Technologies (ICTs) such as computers and the Internet can do for and to students are mixed, perhaps reflecting sociocultural myths in societies around the world (Hollis et al., 2020). In countries like the United States and China, adults are torn between their belief in ICTs’ empowering nature and the anxiety about the risks that ICTs may pose to their children (Livingstone and Bober, 2006; Liu, 2011; Xiao, 2019). Parents value education and recognize the opportunities ICTs have to offer for their children’s development and learning in key subjects such as reading, mathematics, and science (Skryabin et al., 2015). As Latino immigrant parents do in America, Chinese parents expect their children to treat ICTs “seriously” and avoid using them “like a game” (Tripp, 2011, p. 557) too. While ICTs begin to influence children’s learning and development earlier and earlier (Dong, 2018; Dong and Mertala, 2019), parents and teachers are increasingly aware of the limitations they have and are alert to the potential risks ICTs might pose to children and young people (Davies et al., 2019), amongst which are addiction to computer games, inappropriate online content, and a decline in academic performance. In China, academic concerns are probably the primary source of distress for many parents and teachers in middle and high schools (Liu, 2011; Xiao, 2019, 2020). However, such anxiety usually results from the general public’s limited knowledge of and negative media coverage about ICTs in China as well as many other countries around the world (Herring, 2007; Boyd, 2008; Golub and Lingley, 2008; Przybylski and Orben, 2017; Van Rooij et al., 2018), which in turn have a negative impact on how adults regulate adolescent interactions with ICTs. Recent research studies report that the more restrictive adult approaches are, the more likely adolescents are to develop problematic use of the Internet (Xu et al., 2014; Wu et al., 2016).

Adult uneasiness with adolescent engagement with ICTs beyond their supervision is understandable. Social media scholar danah boyd (*sic*) links the disquiet to the panic adults used to hold about outdoor space—young people are vulnerable to the dangers of the outside world and they need protection from adults (2008). Likewise, the Internet is sometimes viewed as a “gateway to harm,” suggesting that “innocent” children must be protected from the dangers of cyberspace such as pornography (Buckingham, 2008), and “dangerous” young people prevented from causing trouble (Valentine and Holloway, 2001). In China, public uneasiness with Internet cafés (wangba in Chinese), a public space often attracting young people from diverse backgrounds to powerful computers connected to the superfast Internet, is not simply about potential harms to the bodies and minds of youths; it has moral implications too (Rao, 2019). For instance, some commentators view Internet addiction as digital opium, analogous to the opium of the late Qing dynasty (1644–1911) that corrupted the moral order of families and societies (Golub and Lingley, 2008). Like the opium in history, the Internet today appeals to “addicts” from all backgrounds, be they poor or rich, of high or low social status, employed or not. Yet it is more difficult to control than the opium, as it is associated with science and technology that also represent progress and civilization.

The addiction analogy, once broadcast to the public, makes people feel uneasy. But the analogy is flawed in at least two ways. First, as Turkle (1995) argued, it attaches more power to the external (wangba) than the internal (students). A more progressive understanding about youths and wangba should thus focus less on the addiction cliché and more on the forces that have kept students so engrossed in wangba and the cyberspace they proffer. Those forces, like the love and emotion we feel toward others, can help us better understand what students are attracted to, what they are missing, and what they need—problems that have deeper meanings (Van Rooij et al., 2018). Second, the argument based on addiction subverts the best possible solution, for it implies that we must get rid of the addictive substance in order to resolve the problem and that, it is the only option for action. But, as Turkle (2011) contended more recently, we are not going to discard the Internet, and the solution the metaphor implies is not going to be the one the society in large will take, even though schools can ban students’ visits to wangba and parents can regulate their children’s access to the Internet. Therefore, constructing students as victims of a harmful substance only makes even more adults feel at a loss and ignores adolescents’ social and informational needs to develop as whole persons.

Young people gather in wangba not merely for access to the Internet, they engage in a wide range of activities that youths normally do in a public place. Just as a personal computer can be used for varied purposes, wangba refer to many things at the same time to different people. To many high school students in this study, they are places where young people can gain access to the Internet outside home and school, play computer games, and socialize with others. To many teachers and parents, they are signs of youth addiction to the Internet, computer games, or anything else they do not want to see. And yet, wangba were easy to find in the three regions where the study took place, particularly near the schools the students attended. As such, it is rather difficult to separately talk about wangba, the Internet, computer games, and addiction the first three may imply.

The research described here is part of a broader study, which employed mixed methods (Johnson et al., 2007; Creswell, 2009; Creswell and Creswell, 2018) to examine how social and educational factors related to adolescent access to and use of ICTs in and out of school, and to understand the meanings and values with which students invested their devices such as computers, mobile phones, and the Internet (Hargittai, 2004, 2010; Hargittai and Hinnant, 2008; Eynon and Geniets, 2016). This manuscript is specifically about adolescent engagement with wangba, which were, and still are, widely available and frequented by students in urban (School Nanshan in Shenzhen), rural (School Hengshan in Hunan), and Tibetan (School Basum in Tibet) regions of China where the fieldwork of the project took place.

The primary focus of this study is on the perplexing relationship between visiting wangba and academic performance, which many adults in China view as causal, as the semi-structured interview data of the research will illustrate. The perceived causal relationship means that visiting wangba can surely *cause* a student’s academic performance to decline, or even *make* them a “worse” person (Liu, 2011; Rao, 2019). Using quantitative data from a survey, the study also seeks to understand more about the relationship by uncovering the “real” effect (as in Modecki et al., 2020) of wangba on academic attainment. Although the primary project did not employ an experimental design, the analysis reported here uses an advanced matching method to draw causal inference from observational data, a technique that is increasingly popular in other disciplines, such as political science (Iacus et al., 2009, 2012, 2019; Ho et al., 2011), where an ideal experiment is not possible to implement, as in this research.

## Materials and Methods

The broader sequential mixed methods research (Teddlie and Tashakkori, 2009; Creswell and Creswell, 2018) first examined how frequently students in different schools visited wangba using the above-mentioned survey (as reported in Table 1). It then interviewed a subset of the survey respondents to understand their attitudes toward and/or lived experiences with wangba and what (perceived) consequences any of their engagement with the technology had on their learning (Selwyn, 2011a,b, 2015).

**TABLE 1:** The first row reports quantified frequency of visits to wangba in the three regions—the higher the value, the more the students visit wangba on average.

	**Shenzhen**	**Hunan**	**Tibet**
Frequency of visits to Internet cafés	0.41	1.65	1.83
Negative views of Internet cafés	2.12	1.80	1.70

*The second row is about student attitudes toward wangba—the higher the value, the more negative attitudes students hold on average toward wangba.*

This research therefore draws on two types of data, one from a paper-and-pencil survey (see also Hargittai and Hinnant, 2008; Murphy, 2008) and the other from in-depth semi-structured interviews (see also Liu, 2009; Micheli, 2016; Xiao, 2019). Prior to the fieldwork, the research project received ethical approval from the School of Education, Durham University. In each school during the fieldwork, permission was sought from teachers who helped distribute the survey to the students they were responsible for. Note that permission from parents was not sought for two reasons. First, all students approached for this research were second year high school students and mature enough to give consent. Second, the majority of the students in rural areas lived in school and their teachers were their guardians. Many students rarely saw their parents throughout the year, and migrant parents usually lived in big cities far away from their children.

Following multiple presentations about the study, the students could ask questions of the researcher, the first author of the paper, before completing the survey in class and with the researcher present to elaborate on any point that was unclear to them. The average age of the participants was 17.4 years when the data were collected and, they all came from the same year group, which means the variation in age was controlled for by design. In total, 698 students from across the three schools, regardless of their residential status (living at home or in school dormitory), filled in the survey.

In addition, at least 15 students were interviewed in each school. The three schools were typical state schools in the three regions, and they all prepared students for the same National College Entrance Exam called Gaokao in Chinese (for more details about the sampling strategy, see Xiao, 2019, 2020). Within each school that resembles many other schools of the same region not selected for the research, at least four classes were selected to represent Arts versus Science, as well as Key versus Ordinary classes, two key variables that are strong predictors of academic performances and opportunities for higher education in China. All students present in the chosen classes completed the survey at the same time. Interviewees were purposively selected from different socioeconomic backgrounds (Teddlie and Yu, 2007), which were approximated by years of parental education and highly correlated with overall (sum of access to the Internet, computers, and mobile phones in and out of school) levels of access to ICTs [see Xiao (2020, p. 263) for a detailed statistical context].

It is worth mentioning that students in all three regions visited wangba, regardless of their overall levels of access to ICTs in school and/or at home. Also, most students in the study lived in school and rarely visited home during term time. Wangba were amongst the most popular places for many students to spend their free time. As schools usually believe time spent in wangba has negative consequences for student learning and adolescent development, they normally ban student access to wangba, even though they are outside and beyond the control of schools. However, young people visit wangba for varied purposes, and their attitudes toward wangba vary from person to person and region to region. As shown in Table 1, Tibetans hold the least negative attitudes toward wangba. Shenzhen students are least likely to visit wangba, but most likely to distain wangba.

### Interviews

#### Depicting Wangba, Defining People

While the condition of wangba is sometimes regarded as an indication of economic development for a town, visiting wangba is often viewed as a sign of a person’s quality (suzhi). Dark, messy, smelly, and crowded, the unpleasant environment in many wangba is due to underdevelopment of Shihong as a town where School Hengshan is located. Implicit in such student perceptions is the view that wangba in bigger cities should be more comfortable than theirs.

In China’s Southern metropolis Shenzhen, Nanshan students also associate wangba conditions with users’ personal qualities. For instance, Sin admitted that most wangba are distasteful. However, he emphasized that one can find pleasant ones in Shenzhen:

It depends on which type of wangba you visit, some bigger ones have better visitors, they would not smoke there; but if you go to those smaller ones, there is nothing you can do about it, cos people there have lower quality.

Although Sin regarded visitors of smaller wangba as having lower quality, he challenged the view that students visiting wangba are “bad.” He treated such stereotyping as outright prejudice. “Following this logic,” continued he, “no student could be counted as a “good” student. To be honest, in my former class, nobody would believe that one had never visited wangba, the best students all went there.”

Student views of wangba in Shihong and Shenzhen reflect their perceptions of socioeconomic development level of the town or city they are situated in. Students in both places agree that the conditions in some wangba are not pleasant, but those in Shenzhen emphasized that it is possible to find better ones in their city, which many Shihong students could only imagine.

But to many adults in Shihong, visiting wangba has a moral connotation. To them, according to Qing, students visiting wangba frequently are not “good” students. Weizhou’s parents do not want him to surf the Internet in wangba. In their view, wangba are not places “good” students ought to visit, and those who frequent wangba are generally speaking not “good” at their studies, or they would become “bad” should they carry on going there. While student views on wangba conditions differ from region to region, their understanding of adult attitudes toward their engagement in wangba is largely consistent—socioeconomic development can improve the material conditions of wangba, but it cannot change the pejorative views of wangba many adults hold.

The above adult views on wangba, once endorsed in media, also affect how students see wangba and themselves (see also Van Rooij et al., 2018). In Shenzhen, some students’ aversion to wangba can be so intuitive that they call it “common sense.” For instance, Xiumeng argued that students visiting wangba are “no good,” for the conditions there are poor. With a computer at home, she did not see any point of going there. Wangba to her are simply places for computer games, which girls like her do not play. Without any time ever spent in wangba, she said her impression of them had never been positive. She then remarked: “It has been internalized since we were little. It’s like your first reaction to cockroaches. You don’t need to experience it. It is intuitive.”

As shown above, both conditions of wangba and discourses surrounding young people’s engagement in wangba are subject to the forces of broader socioeconomic and sociocultural factors, which can change the former, but uphold the latter across regional differences.

#### Monitoring Behavior, Managing Wangba

Parents’ knowledge of wangba influences how they regulate their children’s engagement with wangba and how students view and use them. With parents working in another province as migrant workers for most of his formative years, Yao of School Hengshan in Shihong recalled that his mother returned to specifically monitor his behavior, for he was once fanatical about the Internet. But his mother was not always that effective in her control. What she could do was often left to catching him in wangba. When successful, she normally scolded him, and occasionally beat him. Most of the time, however, the battle between Yao and his mother took the form of quarrels. He confided: “She always insisted wangba were bad for me, but I never felt that!” Unsurprisingly, he kept doing what he wanted to do with the Internet, and simply ignored his father’s admonition conveyed over the phone.

In Shihong, Yao’s parents are not unique in their way of regulating their children’s engagement with wangba. To Wei’s parents, wangba are associated with “only disadvantages, no advantages,” lamented Wei, for “they know nothing about the Internet… all they see is people playing games.” Ting, from the same school, also reported that her parents “believe whatever others have to say about the Internet.”

School Hengshan of Shihong is a typical school in rural China and the majority of the students in the school are children of migrant parents. Common in the student views reported there is the absence of presence—on the one hand, parents are far away, often throughout the year; on the other hand, they want their influence in their children’s education and development, however, impotent, to be felt, if not effective. Both parents and students, again, are too powerless to resist the invisible forces of social machinery—students could not migrate together with their parents and study in host cities, and parents had to leave their children behind to seek better job opportunities in other places than their home town.

Parents knowing more about wangba thought their children should have computers and the Internet at home. Involved in an IT business in Shenzhen, Lu’s father bought her a computer and had it connected to the Internet. He himself visited wangba often for commercial reasons during his time at home in rural Hunan, where home access to the Internet was not common at that time, but his experience in wangba convinced him that he should keep his teenage daughter away from wangba. According to Lu of Shihong, her father did not want her to surf the Internet in wangba, for people there are “complicated” (fuza) and the environment there “messy” (luan).

When it comes to the control of a student’s visit to and experience in wangba, teachers do not differ that much from parents. All three schools in the study ban students’ visit to wangba during weekdays. Hengshan in Hunan and Basum in Tibet even warn their students that anyone who dares to go there would be expelled. Dunzhulaba of School Basum found this policy unacceptable, arguing that appropriate amount of time spent in wangba is okay and his school should only penalize those who use it excessively, for instance, those who break out of the school during weekdays. “Expelling students because they frequent wangba is a fascist rule,” commented the student. “Everybody visits wangba today, even those kids do! We are over 18 and grown up,” he continued. Zaxipubu shared a similar view. He believed high school students are able to discipline themselves well, and the Internet can be of value to their studies. Student views in Tibet, as in those of the other two schools in the study, once again reflect the disconnection between adult views of wangba and what wangba really mean to the adolescents they have a duty of care for.

In School Hengshan, as Qing of Shihong reported, a few students she knew were expelled. The narratives given by those students who were punished can better show how seriously their teachers took the issue. Yao, once caught and then subjected to some disciplinary action, recalled his experience in Hengshan:

Once, we broke out from the school during lunch break in order to surf the Internet in wangba. After we returned, the teacher in charge began to investigate… I eventually confessed the misdeed, but was later asked to write an essay and a note of apology … My parent was also asked to visit the school. The teachers insisted that a parent must come. It is important to inform my parents that I am such a (bad) student in the school, and it is not their fault if I fail — don’t blame teachers if that happens… I then had to read the apology in front of my class. In the essay, I first described what I did wrong, and then listed all the disadvantages of wangba (I could imagine).

Teachers undoubtedly shoulder great responsibilities in the above schools, particularly when parents are migrant workers and/or far away from school. The school policy to ban student visits to wangba appears to represent a crude display of force, but one that can prevent potential risks to the students under their duty of care, risks often beyond their control. This reality thus reveals the vulnerability of schools to many unforeseeable risks in modern societies (Giddens, 1999).

#### Time Spent in Wangba and the Consequences for Learning

As reported above, it is widely held that spending too much time in wangba has negative consequences for learning. Those who have experienced a fall in examination score agree with the view to a certain extent, but they do not necessarily believe that it is the Internet that has the undesirable effect. Yao in School Hengshan, for instance, did not attribute his decline in academic ranking to the technology alone. Upon analyzing his own experience and observation, he was not quite sure if there is a direct link between addiction to the Internet and academic performance, just as some recent studies (Etchells et al., 2016; Przybylski and Orben, 2017; Orben and Przybylski, 2019) report about the link between social media use and young people’s health and wellbeing outside China. He first argued that people around him in the real world—in wangba—posed no risk to him if he managed his own business in cyberspace. Instead, it was those in the virtual world—in games—who affected him as a player. Upon consideration, Yao concluded that what really matters is not the people in the virtual; rather, it is down to himself—if he could discipline himself well, the Internet would do no harm to him at all.

Yao was no longer “addicted” to the Internet and wangba were no longer important to him when he was interviewed—he could better control himself than before, so his exam scores rose again. However, he noted that his performance could have been better had he visited wangba less—his Internet use certainly affected his academic standing. While he realized that his time spent in wangba resulted in a decline in his academic standing, he still performed much better than did his friend who dropped out from school, suggesting that playing with ICTs simply deflated his academic score, but ruined his friend’s academic career. Eventually, he concluded that the effect varies from person to person—while it did not have a devastating impact on him, it had on others around him. Another 17-year old from the same school, Wei, shared Yao’s view and affirmed that there are only benefits associated with wangba if students can control themselves well. As such, this echoes well with findings from other studies that not all students are equally prepared to withstand the effects of online risks (Turkle, 1995) and digital technologies such as wangba are “bad for some teens, not all” (Odgers, 2018; Orben et al., 2019). The differentiated impacts of wangba on different teenagers attest to the fact that some students can still develop agency for themselves–they are not just victims of a harmful technology.

Regarding online benefits, students also differ in how they use wangba to their advantage. In School Hengshan, Guoyu found the Internet so helpful that he could easily find example essays when he really struggled with his own. He revealed that his teacher once asked them to write a report, which he did not know how to even start. Then he went to wangba after school and copied one from the Internet by hand. He justified his decision by pointing to the fact that his teacher did not say students could not copy from the Internet, and that there were simply too many assignments and they were all very difficult. Weizhou did not copy model essays verbatim—he rewrote them according to his memory after he had read them online. He also said homework assignments were too difficult to cope with. If they were easy, stressed Weizhou, he would not have copied them. However, he searched on whatever website that happened to have a search engine. In his words, “I search wherever the home page is.” Yao also used online search engines to find similar essays. But he said he did that usually after he had submitted his own. He maintained that he could learn from others by reading their essays.

The broad social-lingual environment shapes how students learn in wangba too. In Basum where students need to learn Tibetan, Mandarin, and English concurrently (Xiao and Higgins, 2015), wangba provide opportunities for teenagers such as Dunzhulaba to develop interests in music and English. Apart from gaming and chatting, he actively participated in rap forums, particularly those in English. He said most English words he knew were learnt from rap. When he came across a sentence he could not understand, he copied and pasted it in Baidu, the then most famous search engine in China, for a translation/explanation and sometimes even a pronunciation. He regarded the Internet as a better place to learn English than his real classroom. Finally, he emphasized: “It all depends on how you use it.”

Adolescent use of the Internet, according to the above reports, reflects not only what students learn, but also how they are taught in schools. Teachers in the schools studied did not seem to have realized that their students could copy from the Internet the homework tasks they assigned, nor did they teach students how to search resources from the Internet and use them appropriately. As discussed elsewhere (Liu, 2010; Xiao, 2019), the high school curriculum focuses primarily on the high-stake examination, the Gaokao, which limited the amount of time students in the study had at their disposal. In Tibet, adolescent use of the Internet even reveals a linguistic element less visible in the other two schools.

### Survey

#### Experience in Wangba and Academic Attainment

Since all the schools in this analysis have in place some wangba policy and it affects students in varying ways and to varying degrees, it is important to see how experience in wangba relates to academic attainment. In the survey, students were asked about the frequency of their visits to wangba (see Question 13 of the Survey). This variable is converted into a group status indicator for the quantitative analysis, where those who reported “Never before” in terms of experience in wangba are considered “treated,” as if in an experimental design; and those who selected other options such as “Only weekends or holidays,” “Often, even during term time,” “Cannot cope without it,” or “Prefer not to say” form a comparison group for the quasi-experimental design. The outcome is a binary variable called “achieve” or “undera.” The former refers to high academic achievement, where “Above average” or higher in self-reported academic ranking is coded as 1, “Average” or lower as 0. The latter means underachievement, where “Below average” or lower is 1, otherwise 0. Self-reported academic ranking may be a sensitive issue and invalid as a measure of academic attainment in a Western context, but in Chinese schools, just as classes are classified as Key or Ordinary, students are usually acutely aware of where they stand relative to their peers, and the measure is largely valid, as verified in how students answered interview questions and how strongly this variable correlated with other statements relevant to academic performances in Question 11 of the survey (Xiao, 2013). Moreover, other studies conducted in similar contexts in China also employed self-reported academic ranking as key outcomes (see Lau and Leung, 1992; Xiao, 2013; Xu et al., 2014; Xu and Li, 2018). Nevertheless, it would still be better to have had their actual performances in official and high-stake tests, but these were too much to ask for from those teachers and schools who had already helped a lot in facilitating the study. Even with official test results, arguments could still be made that mock exams were not as valid as the real Gaokao, which would not take place until after the end of their third year in high school.

To see the difference in probability of students reporting high achievement or underachievement between the two groups, a logit model is employed to conduct the analysis, with the following stochastic and systematic components: *Y*_*i*_ ∼ *Bernoullie* (π_*i*_) and π_*i*_ = (1 + *e*^−*X*_*i*_β^)^−1^, where *Y*_*i*_⊥*Y*_*j*_ for *i* ≠ *j*, assuming all observations are independent. The log-likelihood function derived from the model is as below:


$$L\left(\pi_{i}|y\right)\propto \prod_{i}^{N}{\left(\pi_{i}\right)}^{y_{i}}{\left(1-\pi_{i}\right)}^{1-y_{i}},$$

$$lnL\left(\pi_{i}|y\right)=\sum_{i}^{N}y_{i}\ln\left(\pi_{i}\right)+\left(1-y_{i}\right)\ln\left(1-\pi_{i}\right),$$

$$=\sum_{i}^{N}-y_{i}\ln\left(1+e^{-X_{i}\beta }\right)+\left(1-y_{i}\right)\ln\left(1-{\left(1+e^{-X_{i}\beta }\right)}^{-1}\right),$$

$$=\sum_{i}^{N}\ln\left(1+e^{\left(1-2y_{i}\right)X_{i}\beta }\right).$$

Following the model specification, simulations are then run in R to calculate the differences in outcomes between the two groups by holding constant all relevant covariates at their median values. The means of 50,000 simulated differences in the probability of students reporting high or low achievement and their 95% confidence intervals are reported in Table 2. The simulated results show that all the confidence intervals in the Achievement column contain 0, suggesting no statistically significant effect of experience in wangba on the probability of students reporting high achievement in each of the three schools. However, when the outcome is Underachievement, those who never visited wangba are significantly less likely to report underachievement than those who did.

**TABLE 2:** Means and their 95% confidence intervals of 50,000 simulated differences in the probability of students reporting achievement or underachievement in each of the three schools studied.

**School**	**Achievement**	**Underachievement**
Basum	−0.1056 (−0.0115, 0.2180)	−0.0926 (−0.1711, −0.0141)
Hengshan	−0.1064 (−0.0115, 0.2220)	−0.1193 (−0.2195, −0.0163)
Nanshan	−0.1028 (−0.0114, 0.2126)	−0.1435 (−0.2785, −0.0176)

However, it is worth noting that the differences reported above assume independence of observations within schools. If we take into consideration the fact that students in the same school have more in common than those from different schools, the uncertainties surrounding those estimates would be more conservative or wider than those reported in Table 2 (Gelman et al., 2012; Xiao et al., 2016), meaning any statistically significant differences we see in Table 2 are likely to be due to chance. Also, the comparisons in Table 2 are between students who are in the middle of various dimensions or covariates used to run the simulations and, the academic ranking variable has 94 missing values, the robustness of the findings is thus questionable.

To address the problems identified above, Amelia (Honaker et al., 2012), an R package, is then employed to impute 10 complete datasets, which have exactly the same observed values but differ in the missing ones. Within each complete dataset, a method called Coarsened Exact Matching (Ho et al., 2011) is used to establish two groups of students who are in the same school, from similar socioeconomic backgrounds, equally motivated to do well in school, and engage with ICTs for about the same range of purposes—measured covariates used to establish the comparison. After matching, the same logit model introduced earlier is implemented to compute the mean differences in outcomes in another R package called Zelig (Imai et al., 2013), which makes it possible to see how strong the relationship really is between experience in wangba and academic attainment. This time, the point estimate for achievement is −0.0463 (−0.2676, 0.1749), and that for underachievement is −0.0354 (−0.1936, 0.1227), showing a random relationship in either direction. Taken together, the results suggest that experience in wangba is not systematically correlated with academic achievement or underachievement, which is consistent with recent findings published outside China on the relationships between screen time and a number of outcomes concerning children and young people (Etchells et al., 2016; Orben and Przybylski, 2019; Orben et al., 2019).

Nevertheless, the quantitative strand of the study is not a real experiment, which requires random allocation of students to treatment or control (Xiao et al., 2017). Instead, it aims to make “fair” comparisons between the two groups (Chalmers, 2014; Jones and Podolsky, 2015; Xiao et al., 2016). Therefore, the findings from the procedures described above are less model dependent and more robust to untenable assumptions and challenges associated with missing data than those reported in Table 2. As in many other studies on young people and new media, detecting causal relationships is challenging but the questions we want to answer are usually causal in nature. This is the case even in large-scale, nationally representative cohort and longitudinal studies (Van Rooij et al., 2018; Orben and Przybylski, 2019; Orben et al., 2019; Orben, 2020). That said, this study has in-depth interview data borne out of the lived experiences of those affected and, the qualitative findings reported earlier not only provide some in-depth domain knowledge that is crucial for the decisions made in later analytical processes, but they also undergird the conclusions from the quantitative strand that has taken robust measures to establish a fair comparison based on observed covariates and deal with any bias associated with missing data.

## Limitations

This analysis suffers from a number of limitations and the conclusion drawn should be treated with caution. First, we used observational data to draw some causal inferences, which is less ideal than a true experimental design. However, randomly allocating the target group of high school students into wangba and non-wangba groups would be impossible to implement, practically and ethically. We made the best use of the data collected and aimed to answer the questions that are causal in nature. A recently published study did show that the conditional independence assumption is largely valid, as is the case for this analysis using matching, and that non-experimental approaches to causal inference can satisfactorily answer such questions in an often more cost-effective and ethical manner and, should play an increased role in socio-educational research (Weidmann and Miratrix, 2020). Second, the primary outcome used for the analysis is self-reported academic standing. It would be better to have test results from standardized tests such as the Gaokao or the mock exams for the Gaokao. But, again, it would be too much to ask for when the research relied upon the good will of the schools and teachers involved, or it would take another year before we could have had access to the data, let alone the challenges associated with data protection and other ethical issues that might bring about. That being said, many research studies rely upon self-reported data (Stavropoulos et al., 2013; Xu et al., 2014; Wu et al., 2016), the validity and reliability of self-reported academic standing may not differ too much from the use of interviews. Finally, the students surveyed came from three schools in three different regions of China. The sample could have been more representative. However, the types of schools chosen for the study were typical state schools in the three regions, and within the schools, key variables such as key versus ordinary classes and arts versus science tracks were considered when students were sampled within the schools. There are many studies about young people’s engagement with digital technologies and many of them are large-scale or even longitudinal in nature (Etchells et al., 2016; Van Rooij et al., 2018; Orben and Przybylski, 2019; Orben et al., 2019). Smaller studies such as this can contribute to the body of knowledge in its unique ways, particularly when they utilize multiple types of data. In this study, for example, a number of decisions made in the quantitative analysis stage were informed by findings from interviews with the students, although the integration of the findings from the two strands could have been made stronger, particularly in how they answered the research questions in a more effectively way.

## Conclusion

Wangba and the narratives about wangba, though largely constructed by adults, have the capability to “script” adolescents (Miller, 2013). The concerns adults repeatedly express to young people clearly exhibit a “technologically deterministic” (Aagaard, 2017) tone—while they ban adolescents’ visits to wangba, they also consider unavoidable the impact on those students with poorer will power, a sign of poor character in the eyes of many. Although schools regard it as vital to implement a strict wangba policy due to various pressures, students also consider it essential to visit wangba for varied purposes.

The concerns surrounding wangba are essentially social, familial, and educational challenges, which are unlikely to be solved through technological interventions alone. Since experience in wangba appears to have little bearing on academic attainment and students need access to both real and virtual worlds in order to mature (Davies, 2017), it might be more desirable than an outright ban if adults adjust to helping them navigate through the virtual as well as the real (Boyd, 2008, 2014) and, if relevant authorities improve wangba conditions so that they become safer and more youth-friendly public spaces outside home and school environments.

## Data Availability Statement

The dataset and its associated R codes can be found in this open repository: https://doi.org/10.7910/DVN/25720.

## Ethics Statement

The research involving human participants was reviewed and approved by the Ethics Committee of the School of Education, Durham University. The students were old enough to give informed consent, and their teachers acting as their guardian gave consent too and helped recruit the students for the study. The research was conducted in accordance with national legislations and institutional requirements.

## Author Contributions

ZX conducted the research, collected and analyzed the data, and authored the manuscript. SH supervised the overall research project, contributed to the conception of the research design, offered critical feedback for earlier drafts of the manuscript, and revised the manuscript after peer review. Both authors contributed to the article and approved the submitted version.

## Conflict of Interest

The authors declare that the research was conducted in the absence of any commercial or financial relationships that could be construed as a potential conflict of interest.

## Publisher’s Note

All claims expressed in this article are solely those of the authors and do not necessarily represent those of their affiliated organizations, or those of the publisher, the editors and the reviewers. Any product that may be evaluated in this article, or claim that may be made by its manufacturer, is not guaranteed or endorsed by the publisher.
