# Supplementary material for: Of Young People and Internet Cafés
Source: Front Psychol. 2021 Sep 16;12:603992. doi: 10.3389/fpsyg.2021.603992 (PMC8481679; doi:10.3389/fpsyg.2021.603992)
Supplement: Supplementary file 1 [file Data_Sheet_1.PDF]

*** 3. Year of birth 丨你是哪年出生的？**

- ◯ 1992
- ◯ 1993
- ◯ 1994
- ◯ 1995
- ◯ 1996
- ◯ I prefer not to say 丨我不想说

Other (please specify)丨其它（请说明）

*** 4. Ethnicity 丨民族**

- ◯ Tibetan 丨藏
- ◯ Han 丨汉
- ◯ Other 丨其它

*** 5. Are you on an arts or science track 丨你读文科还是理科?**

- ◯ Arts 丨文科
- ◯ Science 丨理科
- ◯ Neither丨没有分科

*** 6. Is your class high attaining relative to others in your school 丨你们班是重点班吗?**

- ◯ Yes 丨是
- ◯ No 丨不是

Other (please specify) 丨其它 (请说明)

*** 7. What do your parents do for living? (If you prefer not to reveal this information, please type "N/A") 丨你父母是做什么的？ (如果你不想透露此信息，请填"N/A")**

Mother 丨母亲:

Father 丨父亲:

*** 8. Your parents' highest academic qualification 丨你父母的最高学历**

	Mother 丨母亲	Father 丨父亲
No formal education at all 丨没有受过正规教育	☐	☐
Primary school 丨小学	☐	☐
Junior secondary school 丨初中	☐	☐
Senior secondary school 丨高中	☐	☐
Higher education with or without a first degree 丨大学(大专或本科)	☐	☐
Graduate or higher degree 丨研究生或更高学位	☐	☐
I prefer not to say 丨我不想提供此信息	☐	☐

*** 9. Where do you mainly live during school terms丨上学期间你主要住在什么地方？**

- ◯ At home 丨家里
- ◯ In school dormitory 丨学校宿舍
- ◯ Elsewhere 丨其它地方

*** 10. Academically speaking, where do you stand in your class (overall performance) 丨你的学习表现在班上的大概排名**

- ◯ Top 10%丨前10%
- ◯ Above average丨中上
- ◯ Average丨中等
- ◯ Below average丨中下
- ◯ Bottom 10% 丨最后10%
- ◯ I prefer not to say丨我不想说

*** 11. Please rate the following statements (the higher the score, the more it represents you) | 请为以下表述评分（分值越高就越体现你的状态）**

	1	2	3	4	5
(1) I hope to do well in school this year | 我希望今年学得很好	○	○	○	○	○
(2) I believe that I can do well if I try hard | 我相信，如果我努力，我可以学得很好	○	○	○	○	○
(3) It is important to me that I do well in school | 成绩好对我来说很重要	○	○	○	○	○
(4) When I am taught something that doesn't make sense to me, I spend time trying to understand it | 当老师教的内容很难懂时，我会花时间去弄懂它	○	○	○	○	○

*** 12. Your level of access to ICTs (multiple options possible) | 你对这些技术的使用情况（可多选）**

	None | 没有任何一项	Computer | 电脑	Mobile Phone | 手机	Internet | 互联网
(1) Which items do you have personalised access to | 哪些设备可供你个人使用？	☐	☐	☐	☐
(2) Which items do you have access to at home | 你在家里可以使用哪些设备？	☐	☐	☐	☐
(3) Which items can you use at school | 你在学校可以使用哪些设备？	☐	☐	☐	☐

*** 13. Your visit to Internet cafés | 你去网吧的情况**

- ○ Never before | 从没去过
- ○ Only weekends or holidays | 只有周末或放假的时候去
- ○ Often, even during term time | 学期的时候也经常去
- ○ I can't cope without Internet cafés | 没网吧的话这日子没法过了
- ○ I prefer not to say | 我不想告诉你

*** 14. How many mobile phones have you had for your own use | 你已用过几部属于自己的手机？**

- ○ 0 | 没用过
- ○ 1-3 | 1－3部
- ○ 4-6 | 4－6部
- ○ Above 7 | 7部以上

*** 15. On average, how much per month do you spend on mobile phone bills | 你平均每月花多少钱在手机费上？**

- ○ 0 | 零
- ○ Below 30 Yuan | 30元以下
- ○ 30－90 Yuan | 30－90元
- ○ 90-150 Yuan | 90－150元
- ○ 150－250 Yuan | 150－250元
- ○ Above 250 Yuan | 250元以上

*** 16. Usually, what do you use the Internet for (multiple options possible) | 你一般用互联网做什么（可多选）？**

- [ ] Online banking | 网络银行
- [ ] Visiting social networking sites such as Renren | 访问人人等社交网站
- [ ] Chatting online | 聊天
- [ ] Downloading videos or music | 下载视频或音乐
- [ ] Receiving and sending emails | 收发电邮
- [ ] Playing online games | 玩网络游戏
- [ ] Keeping up with news | 看新闻
- [ ] Looking for job opportunities | 找工作
- [ ] Meeting international friends | 会国际友人
- [ ] Running a business | 做买卖
- [ ] Editing or sharing photos | 编辑或分享照片
- [ ] Reading novels online | 读网络小说
- [ ] Researching products I'd like to buy | 查询购物信息
- [ ] Writing my own blog | 写日志

Other (please specify) | 其它（请说明）

____

*** 17. How do you use ICTs for your study (multiple options possible) | 在学习上，你是如何使用这些设备的（可多选）？**

- [ ] Writing homework on computer | 用电脑写作业
- [ ] Making a slide presentation such as PowerPoint | 准备电子演说文档，如微软的PowerPoint
- [ ] Searching online for information related to my study | 在网络上查找与学习有关的信息
- [ ] Logging on to school website to get information about school work | 登录学校网站查询与学习有关的信息
- [ ] Communicating with friends about school work | 跟朋友交流与学习有关的信息
- [ ] Usually, I don't use ICTs for school work | 我一般不因为学习而使用这些设备

Other (please specify) | 其它（请说明）

____

*** 18. When you encounter difficulties while using ICTs, where do you get help (multiple options possible) | 当你在使用这些技术的过程中遇到困难时，你可以从哪里得到帮助（可多选）？**

- [ ] I try to figure it out for myself | 我尝试自己去弄懂它
- [ ] Friends | 朋友
- [ ] Teachers | 老师
- [ ] Parents | 父母
- [ ] I look on the Internet | 我上网找解决办法
- [ ] Nobody available to help | 没有人可以帮我

Other (please specify) | 其它 (请说明)

____

*** 19. How restrictive are the conditions you face (e.g. those from either your parents or teachers) in using the following ICTs | 你在使用以下信息技术时面临多大来自比如父母或老师的限制？**

	No access or no opinion 没有接触或没有看法	Very restrictive 很受限制	Fairly restrictive 有一定限制	Not restrictive at all 一点限制也没有
Internet café | 网吧	◯	◯	◯	◯
Mobile Phone | 手机	◯	◯	◯	◯

*** 20. To what extent do you agree with the following statements | 你在多大程度上同意以下看法？**

	Strongly disagree | 很反对	Largely disagree | 基本反对	Largely agree | 基本赞成	Strongly agree | 很赞成	No opinion 没意见
(1) Internet cafés are significantly detrimental to students like me | 网吧对我这样的学生来说是极其有害的	◯	◯	◯	◯	◯
(2) Computer games have severely affected my academic performance | 电子游戏严重地影响了我的学习	◯	◯	◯	◯	◯
(3) My use of ICTs has a negative impact on my relationship with my parents | 使用这些设备破坏了我与父母之间的关系	◯	◯	◯	◯	◯
(4) Most information I find online can be trusted | 大部分网络上的信息是值得信赖的	◯	◯	◯	◯	◯
(5) I am very concerned about my privacy online | 我很担心我的网络隐私	◯	◯	◯	◯	◯
(6) With a mobile phone of my own, I can build a closer relationship with my friends | 自己有手机可以更好地建立友情	◯	◯	◯	◯	◯

21. If you would like to take part in a follow-up interview, please leave your contact details below. I will be in touch with you shortly | 如果你愿意跟我面谈，请留下你的联系方法，我会尽快与你联系。

QQ ____

Phone No. | 电话号码 ____

Email | 电邮 ____

Other | 其它 ____

This is the end of the survey. Your answers to the questions have been received with appreciation | 你已经回答了该问卷的所有问题，非常感谢你的时间！
